# Effect of Probiotics on Sperm Quality in the Adult Mouse

**DOI:** 10.1007/s12602-024-10388-z

**Published:** 2024-10-23

**Authors:** Ana Sanchez-Rodriguez, Ingrid I. D. Idrovo, Rocío Villafranca, Nerea Latorre, Juan Antonio Rielo, Ane Laburu, Sandra Nieto-Román, Daniel Heredia, Rubén González, Virginia García-Cañas, Diego Laxalde, Carolina Simó, David R. Vieites, Eduardo R. S. Roldan

**Affiliations:** 1 Department of Biodiversity and Evolutionary Biology, Museo Nacional de Ciencias Naturales (CSIC), Calle José Gutierrez Abascal 2, 28006 Madrid, Spain; 2 Department of Biogeography and Global Change, Museo Nacional de Ciencias Naturales (CSIC), Calle José Gutierrez Abascal 2, 28006 Madrid, Spain; 3 BioCoRe S. Coop. Calle Primitiva Gañan 11, 28026 Madrid, Spain; 4 Molecular Nutrition and Metabolism, Institute of Food Science Research (CSIC), Calle Nicolás Cabrera 9, 29049 Madrid, Spain; 5 Department of Ecology and Marine Resources, Institute of Marine Research (CSIC), Rúa Eduardo Cabello 6, 36208 Vigo, Spain

**Keywords:** Probiotics, Sperm evaluation, *Faecalibacterium duncaniae* A2-165, *Lacticaseibacillus rhamnosus* GG, Vivomixx®

## Abstract

The administration of probiotics for the treatment of different diseases has gained interest in recent years. However, few studies have evaluated their effects on reproductive traits. The objective of this study was to examine the effect of two mixtures of probiotics, a commercial probiotic (Vivomixx®) and a mix of *Lacticaseibacillus rhamnosus* GG and *Faecalibacterium duncaniae* A2-165, on sperm quality in a mouse model. Adult male mice (8 months old) were used for two experimental and one control groups (*n* = 5 each). The probiotics or physiological serum (control) was administered orally, twice a week, during 5 weeks. Sperm were collected from the cauda epididymis, and their total number, motility, kinematics, morphology, and acrosome integrity were assessed in recently collected samples and after a 60-min in vitro incubation. Results showed a higher percentage of normal sperm in both experimental groups, with fewer head abnormalities than in the control. Differences were found among groups in the morphometry of sperm heads, being more elongated in mice treated with probiotics. Sperm from probiotic-treated mice showed similar total motility when compared to the controls, although the proportion of progressively moving sperm and their vigor of motility were lower. Sperm swimming descriptors were measured with a CASA system. Velocity parameters were similar among groups whereas linearity was higher in mice treated with the commercial probiotic. These results suggest that the administration of probiotics may increase the proportion of sperm with normal morphology and lead to modifications in sperm head shape that may enhance sperm swimming. Studies using a longer administration period would be useful in further characterizing the effect of these probiotic mixtures on sperm quality and fertilization capacity.

**Supplementary Information:**

The online version contains supplementary material available at 10.1007/s12602-024-10388-z.

## Introduction

The World Health Organization (WHO) and the Food and Agriculture Organization (FAO) define probiotics as living microorganisms (yeast and bacteria) that deliver a health benefit to the host when administered in a suitable amount [1]. Probiotics can balance gut microbiota, which regulates host metabolism, immunity, and endocrine function [2]. To develop their benefits, they must be in adequate quantity and reach the distal intestine alive. Once in the intestine, due to their metabolism, microorganisms produce proteins, amino acids, and short-chain fatty acids such as propionate, acetate, and butyrate, which preserve intestinal integrity and diminish intestinal inflammation [3]. At the intestinal level, probiotics are beneficial in several species. In humans, they reduce child diarrhea treated with antibiotics [4]. In young ruminants, they promote optimal maturation of rumen microflora [5]. In stressed calves, in which *Lactobacillus* population decreases, they mitigate gut microbiota shifts and decrease newborn diarrhea [6]. In piglets and poultry, probiotics reduce the risk of diarrhea caused by pathogens [6].

The effects of probiotics are not restricted to the intestine. They also reach other organs and systems, which has led to their use in the treatment of diseases, including cardiovascular diseases [7], hyperlipidemia [8], diabetes [9], and hypertension [10]. However, studies concerning the relationship between the microbiome and the possible role of probiotics in treating infertility or their effects on mammalian reproduction are scarce [11]. There is, therefore, the need to investigate the possible role of probiotics concerning the interaction between microbiome and fertility, aiming to improve male and female reproductive function [12]. To this end, in vivo studies are needed to characterize various aspects of probiotic function, such as appropriate strains to use and their dosage, proper administration, and duration of treatment before they can be considered viable for improving reproductive traits.

The most frequently used microorganisms in probiotic products are species of *Lactobacillus* and *Bifidobacterium*, but species of *Saccharomyces*, *Streptococcus*, and *Escherichia* are used too [13]. Some commercial probiotics are a mixture of several strains. For example, Vivomixx® is a multi-strain probiotic that, according to the manufacturer, contains eight strains of live bacteria (450 billion bacteria per sachet; strain composition is detailed in the “Materials and Methods” section), and such mix appears to be more effective than probiotics formulated with one strain because of its higher total concentration [14]. There are studies of new strains as next-generation probiotic candidates, such as *Faecalibacterium prausnitzii*, a significant component of the gut microbiota, reported to be in low quantity in many intestinal disorders, diabetes, psoriasis [15], or kidney diseases [16]. *F. prausnitzii* A2-165 strain has been the most exhaustively studied and has been recently reclassified and assigned to a new species, *Faecalibacterium duncaniae* sp. nov. [17]. These Gram-positive bacteria produce energy used by the intestinal epithelium and metabolites (i.e., butyrate, salicylic acid) that help maintain intestinal health, modulating the inflammatory process [15, 18]. However, its culture is complex as it needs strict anaerobiosis to grow [19] and its formulation as a probiotic is very challenging.

In reproduction, studies in females have mainly focused on critical stages such as gestation, breastfeeding (with their application to the treatment of mastitis [20, 21]), and weaning [22]), and on the effects on genital tract microbiome and possible infectious and inflammatory processes [12, 23]. In males, the gut microbiota has been found to influence the development and integrity of the blood-testis barrier, possibly having endocrine [24] and testicular immunity roles [2]. It has been suggested that gut microbiota is related to the testicular microbiome [11]. In this sense, bacteriospermia has always been thought to exist only in ejaculates from individuals with infections; however, there is evidence for the appearance of several species of bacteria (i.e., *Escherichia* spp., *Staphylococcus* spp., *Streptococcus* spp., *Enterococcus* spp.) also in healthy men, which raises the question of the possible role of the testicular microbiome in male fertility and infertility [25]. On the other hand, some studies have investigated the effect of probiotic administration on sperm quality and male fertility. In asthenozoospermic and oligoasthenoteratospermic men, the administration of probiotics resulted in an improvement in sperm motility, sperm count, percentage of normal sperm, and DNA fragmentation [26–28]. *Lacticaseibacillus rhamnosus* (formerly, *Lactobacillus rhamnosus*) was investigated in dogs and it was found to improve sperm viability, morphology, kinematics, and acrosome integrity [29]. *L. rhamnosus* administered together with *Bifidobacterium longum* in zebrafish also increased sperm concentration and motility [30, 31]. Moreover, in rats fed with a high-fat diet [32] and in obesity-induced male mice [33], where the sperm quality had decreased due to the obesity, probiotics improved motility and kinematics of sperm.

The objective of the present study was to examine the influence of two probiotic formulations on sperm traits of reproductively mature mice. One of the formulations was a mixture of *Lacticaseibacillus rhamnosus* GG and *Faecalibacterium duncaniae* A2-165 whereas the other was a commercial multi-strain probiotic (Vivomixx®).

## Materials and Methods

### Animal Management

Adult male mice, *Mus musculus*, 8 months old, of the C57BL/6JOlaHsd strain, were used to study the effects of probiotic supplementation on sperm quality. Animals were kept in our animal facilities in individual cages with free access to commercial food and water and maintained under controlled conditions of photoperiod (14-h light, 10-h darkness) and temperature (22–24 °C). All animal procedures and handling followed the Spanish Animal Protection Regulation RD53/2013 and the European Union Regulation 2010/63 and had the approval of CSIC’s ethics committee and the Comunidad de Madrid (28,079–47-A).

### Experimental Design

Fifteen male mice were divided into three groups: (1) control (*n* = 5), which received physiological serum; (2) group V (*n* = 5), supplemented with a commercial probiotic (Vivomixx®, SC Probio Mendes SRL, Switzerland); and (3) group A (*n* = 5), receiving *L. rhamnosus* GG and *F. duncaniae* A2-165. Treatments lasted 5 weeks, which was designed to cover a period equivalent to the ~ 30 days of a spermatogenic wave (of which ~ 14 days are for spermiogenesis) [34–36] and the ~ 8–10 days of transit along the epididymis [37].

### Description of Probiotics

The Vivomixx® probiotic contains, according to the manufacturer (https://www.vivomixx.eu/en/about-vivomixx/), *Streptococcus thermophilus* NCIMB 30438, *Bifidobacterium breve* NCIMB 30441, *Bifidobacterium longum* NCIMB 30435 (reclassified as *B. lactis*), *Bifidobacterium infantis* NCIMB 30436 (reclassified as *B. lactis*), *Lactobacillus acidophilus* NCIMB 30442, *Lactobacillus plantarum* NCIMB 30437, *Lactobacillus paracasei* NCIMB 30439, and *Lactobacillus delbrueckii* subsp. *bulgaricus* NCIMB 30440 (reclassified as *L. helveticus*). *L. rhamnosus* GG (nu-trish® LGG®, Chr Hansen, Denmark) was grown on Man-Rogosa-Shape Agar (MRS, Oxoid, Hampshire, England) and incubated at 37 °C and 5% CO_2_ for 10 h. Cells were harvested by centrifugation (4000 × g for 10 min), and the bacterial pellet was resuspended in MRS at a density of 10^11^ colony forming units (CFU)/mL, freeze-dried, and stored at − 20 °C until used for animal trials. *F. duncaniae* A2-165 (DSM 17677) was cultured at 37 °C in an anaerobic chamber (90% N_2_, 5% CO_2_, and 5% H_2_) in a Gifu Anaerobic Medium (GAM, Nissui Pharmaceuticals, Tokyo, Japan) supplemented with 40 mM sodium acetate and 3.5 g/L inulin for 72 h. The culture medium was left in the anaerobic chamber for at least 24 h before being used to remove all traces of oxygen. Cells were harvested by centrifugation (4000 × g for 10 min), and the bacterial pellet was resuspended at 10^10^ CFU/mL in a solution containing 7% inulin, 0.13% L-cysteine and 5.5 mM riboflavin [38]. The suspension was freeze-dried and stored at − 20 °C upon freezing with liquid nitrogen until used for animal trials.

### Administration of Probiotics

Probiotics or physiological serum was administered orally twice a week using an oroesophageal tube to ensure proper administration. Vivomixx® probiotic (group V) was administered in a 100 µL-suspension containing ~ 10^10^ CFUs, whereas probiotics in group A were administered in 100 µL- and 200 µL-suspensions containing ~ 3 × 10^9^ CFU and ~ 10^9^ CFU, respectively.

### Sperm Collection

Animals were sacrificed by cervical dislocation and weighed with a precision balance (Sartorius AG, Göttingen, Germany). Testes were dissected and weighed. The relative test size (RTS) was calculated using the formula: testes mass / (0.031 × body mass^0.77^) [39]. Spermatozoa were collected from caudae epididymides as previously described [40]. Briefly, caudae epididymides were dissected, placed in a Petri dish containing 1 ml of modified Tyrode’s medium with Hepes (mT-H), and cut several times with fine scissors to allow sperm to swim out into the medium for 10 min at 37 °C. The tissue was discarded and the remaining volume was measured. The medium mT-H contained 131.89 mM NaCl, 2.68 mM KCl, 0.49 mM MgCl_2_·6H_2_O, 0.36 mM NaH_2_PO_4_·2H_2_O, 20 mM Hepes, 1.80 mM CaCl_2_, 5.56 mM glucose, 5 µg/ml phenol red, 50 µg/ml kanamycin, and 4 mg/ml bovine serum albumin.

### Sperm Evaluation

Sperm concentration was assessed using a Neubauer chamber. Total volume was calculated taking into account the volume measured after sperm swim out. Sperm concentration was then adjusted to 20 × 10^6^ sperm/ml in mT-H for further assessments. Sperm parameters were examined soon after collection and after 60 min of incubation in mT-H. Percentage of total and progressive sperm motilities and the quality of motion (ranging from 0 to 5, being 0 immotile sperm and 5 maximum vigor) were assessed using a phase contrast microscope. A sperm motility index (SMI) was calculated using the equation: [((Quality of motility × 20) + Total motility) / 2].

Sperm kinematics were assessed using a computer-assisted sperm analysis (CASA) system (Sperm Class Analyzer, SCA v.6.2, Microptic, Barcelona, Spain) [40]. Parameters measured were VCL (curvilinear velocity, µm/s), VSL (straight-line velocity, µm/s), VAP (average path velocity, µm/s), STR (straightness, STR = VSL/VAP), LIN (linearity, LIN = VSL/VCL), WOB (wobble, WOB = VAP/VCL), ALH (amplitude of lateral head displacement, µm), and BCF (beat-cross frequency, Hz).

Sperm preparations were made from each individual by mixing an aliquot of sperm suspension with an eosin-nigrosin solution (Sigma-Aldrich, Madrid, Spain) and smeared. Then, smears were stained with Giemsa (Sigma) as previously described [41]. Sperm were examined using light microscopy, counting the number of sperm with pale, uniform staining of the post-acrosomal region (live sperm) and dark post-acrosomal staining (dead sperm). Sperm abnormalities were also quantified in these preparations, and classified according to alterations in the head, midpiece, or principal piece.

The percentage of acrosome integrity was evaluated under phase contrast microscopy, in samples fixed with 2% glutaraldehyde (v/v) (BDH, Madrid, Spain) in 0.165 M sodium cacodylate/HCl (BDH).

The nuclear morphology was examined in sperm fixed in 2% glutaraldehyde in 0.165 M sodium cacodylate/HCl and stained with Hoechst 33,258 (Sigma) to identify nuclei. A working solution was prepared by mixing 3 µl of Hoechst 33,258 stock solution (6 µg/ml) and 497 µl of 1 × PBS, pH 7.4 (Gibco, Madrid, Spain). A volume of 4 µl of the solution was added to 10 µl of fixed sperm. After an incubation of 3 min in the dark, 5 µl of sperm suspension were placed between a slide and a coverslip (22 × 22 mm), applying light pressure with a filter paper to remove excess liquid. Samples were observed and photographed using a 100 × immersion objective. An Eclipse 50i microscope (Nikon, Tokyo, Japan) with Plan-Fluor optics and a DS5 camera (Nikon, Tokyo, Japan) were used for digitization. Sperm were photographed using the NIS-Elements software (Nikon) along with a 10-μm scale for calibration. Each sperm was captured in phase contrast and using a UV-2A filter (Nikon), with excitation and emission of fluorescence in blue (330–380 nm). The phase contrast images were taken to observe and establish a visual relationship between the morphology of the sperm head and the nucleus, while the images captured in fluorescence were used for nuclear analysis. A minimum of 50 sperm heads were analyzed per sample. Analyses were carried out with the ImageJ plugin “Nuclear_Morphology_Analysis_1.20.0_standalone” software [40] in stained samples. This method measures the interior angle at every point around the shape’s perimeter and generates an angle profile. The median profile, constructed by taking the median of the angles at each point, generates a consensus nucleus from all the analyzed images (Fig. S1) [42]. Standard nuclear measurements were also obtained (Fig. S2).

### Statistical Analysis

Data were analyzed using GraphPad Prism 9 Software (Dotmatics, CA, USA). One-way ANOVA with Tukey’s *post-hoc* test assessed significant differences between treatment groups in body and testes masses, RTS, total number of spermatozoa, and sperm abnormalities. Two-way ANOVAs of sperm motility and kinematics, viability, and acrosome integrity were done to determine significant differences between treatment groups and times of sampling. All variables are shown as means ± SEM.

## Results

### Comparison of Body, Testes, and Relative Testes Mass

Weights of body and testes were assessed in control and experimental animals. There were no significant differences between the control and the two groups receiving different probiotics in body mass (control, 30.9 ± 1.3 g; group V, 33.0 ± 1.3 g; group A, 31.5 ± 1.4 g), testes mass (control, 0.22 ± 0.005 g; group V, 0.23 ± 0.004 g; group A, 0.22 ± 0.01 g), or the calculated relative testes mass (control, 0.50 ± 0.01; group V, 0.40 ± 0.11; group A, 0.50 ± 0.01) (Fig. S3).

### Sperm Parameters

The analysis of sperm parameters in freshly collected samples showed that sperm concentration appeared to be higher in males of group A in relation to controls, although there were no significant differences between them (control, 36.4 ± 4.9 × 10^6^ sperm/ml; group V, 33.6 ± 3 × 10^6^ sperm/ml; group A, 40.3 ± 2.1 × 10^6^ sperm/ml). Similarly, when examining the total number of sperm, there were no statistically significant differences between controls and the two groups that received probiotics (control, 21.6 ± 2.7 × 10^9^ sperm; group V, 20.2 ± 1.8 × 10^9^ sperm; group A, 24.2 ± 1.3 × 10^9^ sperm) (Fig. S4).

**Fig. 1:**
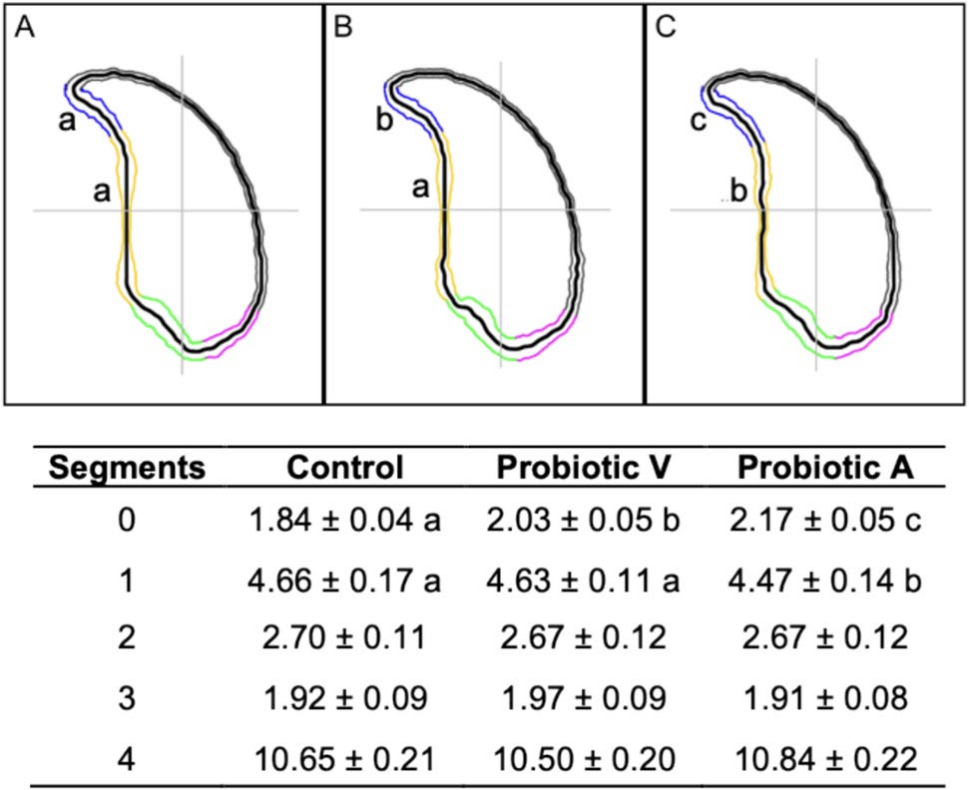
Consensus mouse sperm nuclei of controls and each treatment group, and overlap showing the differing regions. **A** Control. **B** Probiotic V. **C** Probiotic A. Values of different segments (µm) are presented as means ± SEM. Different letters (a, b, c) in the panels and in the same row indicate significant differences between experimental groups (*p* < 0.05)

The results of sperm nuclear morphology analysis showed significant differences in segment 0 in both probiotics groups and in segment 1 in group V compared to control (*p* < 0.05), corresponding with the hook of the sperm head (Fig. 1). Furthermore, the interquartile range (IQR), which indicates the dispersion of data, showed greater dispersion at position 350, that corresponds to segment 3 (Fig. S5). The comparison of angle profiles of sperm nuclei showed no significant differences (Fig. S6).

**Fig. 2:**
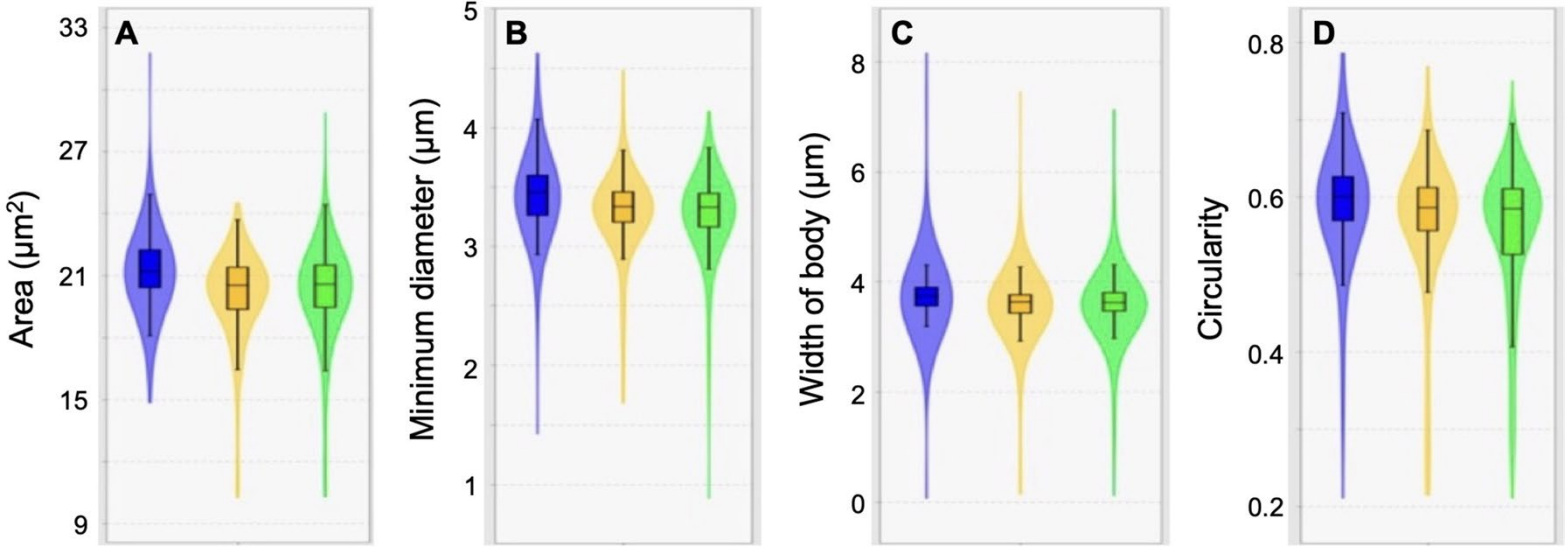
Size and shape measurements of spermatozoa from controls and two groups treated with different probiotics. Shown are comparisons with statistically significant differences. See Table S1 for all comparisons. **A** Area of nucleus. **B** Minimum diameter. **C** Body width. **D** Circularity. Blue, control; yellow, group V; green, group A

Sperm nuclei of control individuals exhibited significantly higher areas than groups supplemented with probiotics (control, 21.21 ± 0.17 µm^2^ *vs.* group V, 20.08 ± 0.16 µm^2^ and group A, 20.28 ± 0.16 µm^2^). The minimum diameter of sperm nuclei was also higher in controls than in experimental groups (control, 3.43 ± 0.03 µm *vs.* group V, 3.28 ± 0.03 µm and group A, 3.26 ± 0.03 µm), as well as in body width (control, 3.79 ± 0.07 µm *vs*. group V, 3.56 ± 0.05 µm and group A, 3.60 ± 0.05 µm). The circularity showed higher values in control (0.59 ± 0.01) than in group A (0.55 ± 0.01) (Fig. 2). The other parameters did not show significant differences between groups (Table S1).

**Fig. 3:**
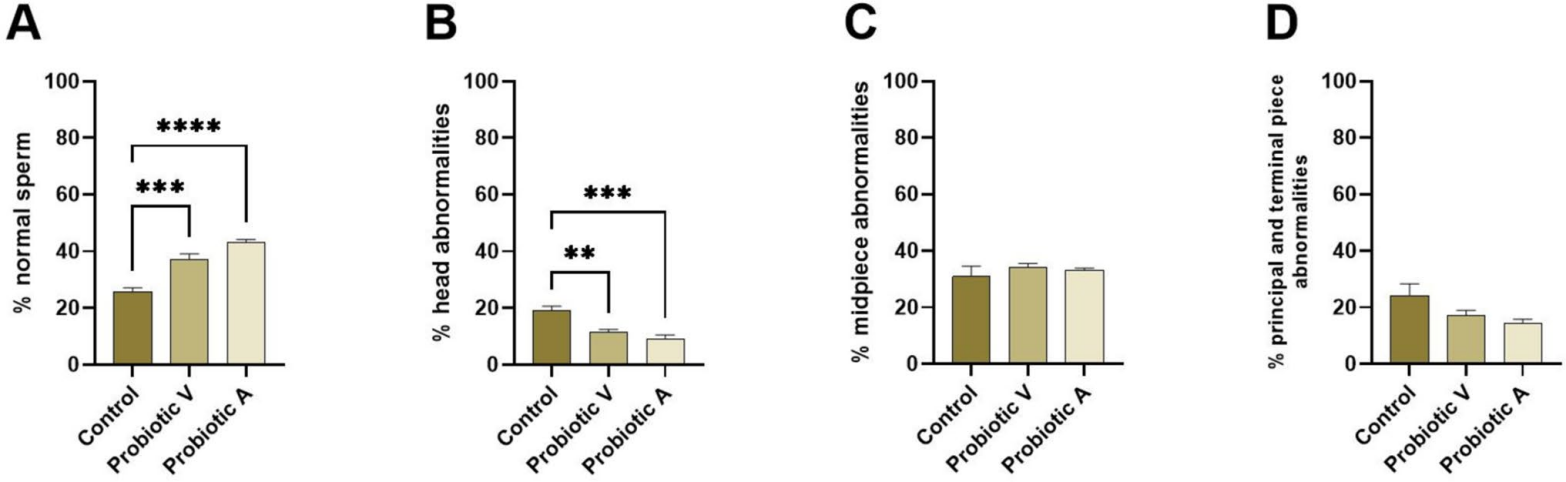
Effect of probiotics on sperm abnormalities in adult mice. **A** Percentage of normal sperm. **B** Percentage of head abnormalities. **C** Percentage of midpiece abnormalities. **D** Percentage of abnormalities in the principal and terminal pieces. *p < 0.05; **p < 0.01; ***p < 0.001; ****p < 0.0001. Data are means ± SEM

Sperm morphology was analyzed to quantify the proportion of sperm with abnormalities in the head, midpiece, or principal piece and, in turn, the proportion of normal sperm. A statistically significant increase in the proportion of normal sperm was observed in mice supplemented with probiotics in comparison to the group that did not receive probiotics (Fig. 3). Analyzing the percentages of sperm abnormalities, the differences relate to a significant decrease in head abnormalities in both probiotic groups when compared to controls. There was also a trend for a decrease in abnormalities in the principal piece in group A compared to the control, but the difference did not reach significance (*p* = 0.06).

The percentage of intact acrosomes in freshly collected sperm cells was similar between controls and the two experimental groups (control, 55.8 ± 4.6%; group V, 57.0 ± 3.6%; group A, 58.5 ± 4.7%; *p* > 0.05).

**Fig. 4:**
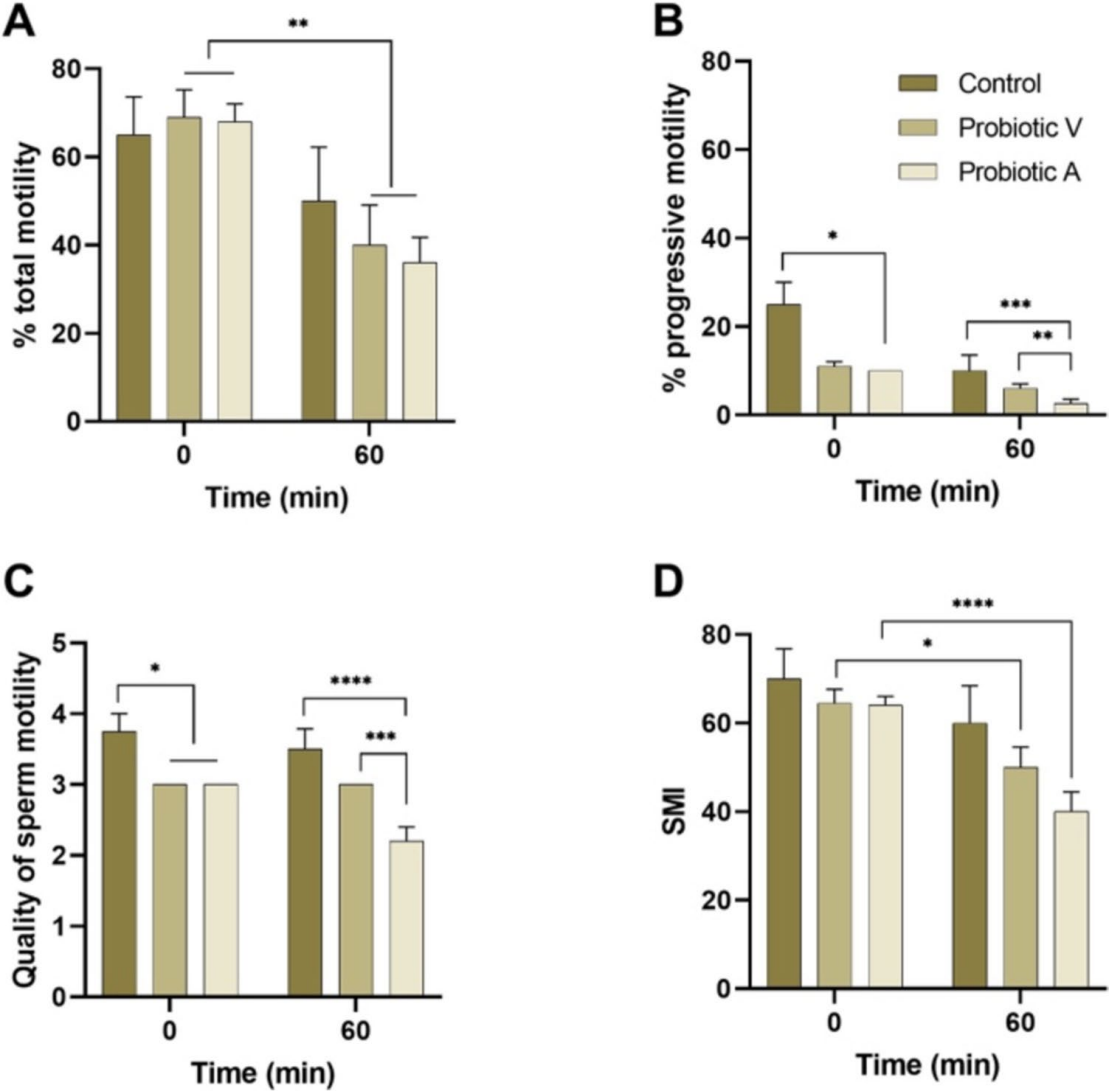
Effect of probiotics on sperm motility in adult mice. **A** Percentage of total motility. **B** Percentage of progressive motility. **C** Quality of motion. **D** SMI, sperm motility index. *p < 0.05; **p < 0.01; ***p < 0.001; ****p < 0.0001. Data are means ± SEM

Motility parameters were examined in freshly collected sperm suspended in a modified Tyrode’s medium (mT-H) (Fig. 4). There were no differences between controls and both probiotic groups in percentage of total motility. When progressive motility was analyzed, both group V and group A exhibited a decrease in the percentage of cells with progressive motility, reaching statistical significance in group A. The quality (vigor) of movement was significantly lower in groups V and A compared to the control. Finally, a sperm motility index (calculated to express both the percentage of total motility and quality) showed a trend towards lower values, but the differences were not significant.

**Fig. 5:**
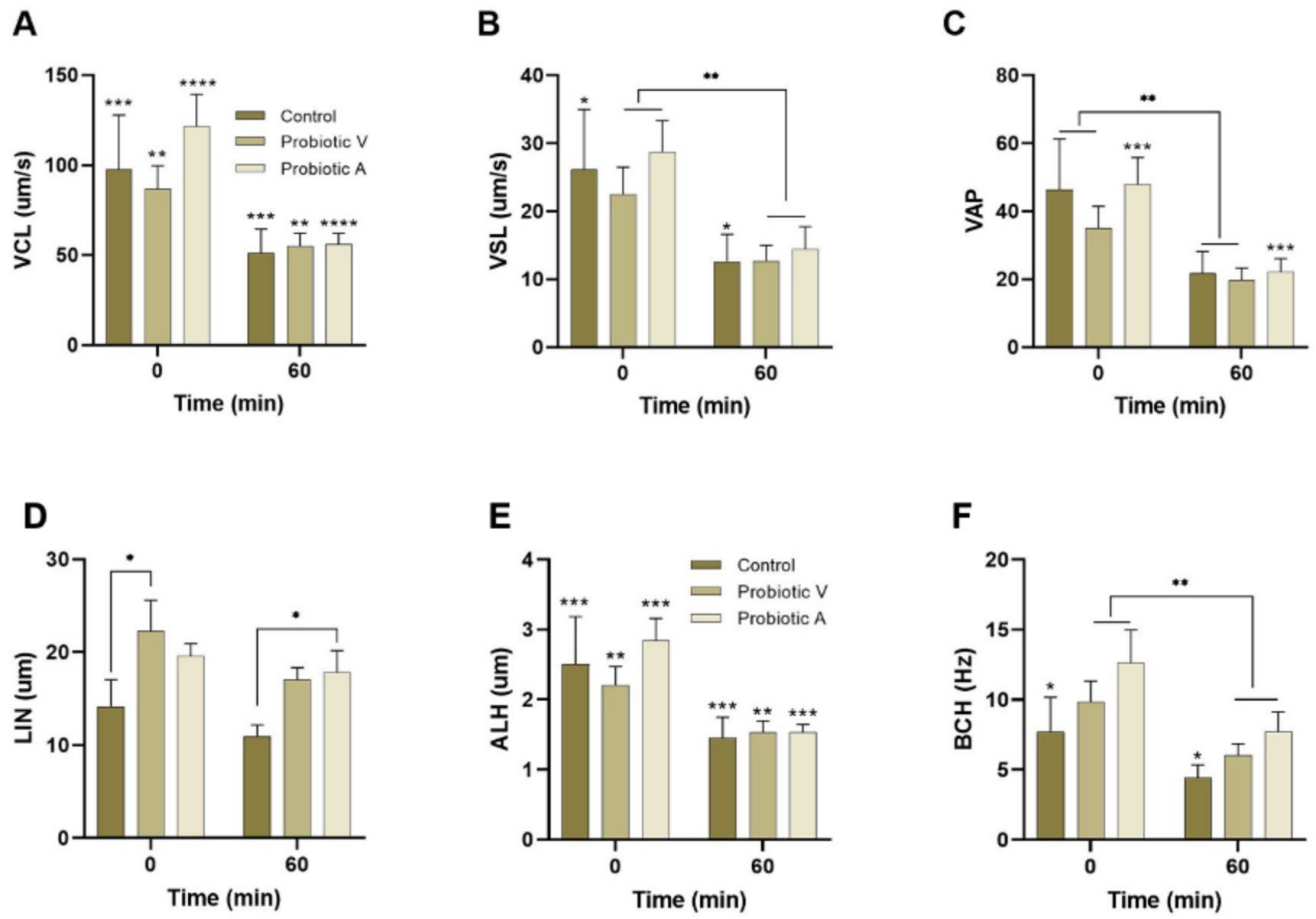
Effect of probiotics on sperm velocities and trajectories measured by CASA in adult mice. Spermatozoa were suspended in a Hepes-containing modified Tyrode’s medium (see “Materials and Methods”) and examined shortly after collection or after 60 min of incubation at 37 °C **A** VCL, curvilinear velocity. **B** VSL, straight-line velocity. **C** VAP, velocity of average pathway. **D** LIN, linearity. **E** ALH, amplitude of lateral head displacement. **F** BCF, beat-cross frequency. Asterisks indicate differences between times (0 min *vs.* 60 min) in the same group (control, probiotic V, or probiotic A). **p < 0.01; ***p < 0.001; ****p < 0.0001. Data are means ± SEM

Sperm kinetics are shown in Fig. 5. Sperm velocities (VCL, VSL, VAP) of the probiotic groups did not differ from those of the control group. Linearity in freshly collected samples was higher in groups V and A than in the control (but only reached significance for group V). Both ALH and BCF were not significantly different between experimental groups and control in freshly collected samples.

### Changes in Sperm Parameters During Incubation of Spermatozoa

Spermatozoa from all males were incubated for 60 min under air in a Tyrode’s medium containing Hepes (mT-H). As shown in earlier studies, this identifies potential changes in spermatozoa resembling those that occur during transit and survival in the female tract before fertilization.

The percentage of intact acrosomes did not change at 60 min when compared to values recorded in freshly collected samples, in controls and the two experimental groups (control, 52.4 ± 11.4%; group V, 59.6 ± 4.9%; group A, 60.6 ± 2.8%; *p* > 0.05).

There was a decrease in the proportion of total sperm motility when spermatozoa were incubated for 60 min, compared to values recorded in freshly collected sperm. However, there were no differences in total motility between the control and the two experimental groups (Fig. 4). Analysis of the percentage of progressively moving sperm after 60 min of incubation revealed a slight decrease in value (although not significant) in relation to control. There were differences between groups, with sperm from males in group A showing lower percentages of progressive sperm than in control and group V (Fig. 4). No changes were observed in the quality of motility over time, but group A was lower in quality in relation to control and group V (Fig. 4). A decrease in SMI was seen after 60 min of incubation in groups V and A (Fig. 4).

Sperm kinetics varied over time. Sperm VCL, VSL, and VAP were lower after 60 min of incubation in relation to time 0, but did not differ between the control group and groups V and A (Fig. 5). Sperm LIN did not decrease over time (Fig. 5) and, as in freshly collected sperm, it was higher in groups V and A than in the control (significant for the former). Sperm ALH and BCF decreased significantly over time. At 60 min, no significant differences were seen in these parameters between the control and the two experimental groups (Fig. 5).

## Discussion

The present study revealed that 5 weeks of supplementation with two different mixtures of probiotics had no negative effects on sperm quality in adult mice and, in fact, may result in an increase in some sperm parameters, such as the percentage of normal spermatozoa and swimming descriptors. To the best of our knowledge, this is the first report of the effects of these two mixtures of probiotics on sperm traits in an adult mouse model.

The first study performed in men with idiopathic oligoasthenoteratospermia was carried out by Maretti and Cavallani [27] after they realized by chance that some men who ingested a commercial probiotic composed of *Lacticaseibacillus paracasei* (formerly *Lactobacillus paracasei*) mixed with prebiotics improved their sperm traits. The authors performed a study evaluating the effect of that probiotic, administered for 6 months, on sperm quality of men with idiopathic oligoasthenoteratospermia, and found a significant improvement in semen volume, sperm concentration, progressive motility, and proportion of normal sperm. Our results partially agree with this study in human patients, since we observed an increase in normal sperm in groups receiving probiotics compared to the control. Another study in asthenozoospermic men [26] examined the effect of the administration for 6 weeks of two strains with antioxidant properties (*L. rhamnosus* CECT8361 and *Bifidobacterium longum* CECT7347) on sperm parameters. Sperm motility and sperm DNA integrity improved, while other sperm parameters were not enhanced, similar to our findings. A subsequent study in infertile men [28] examined the effect of probiotic combination (*Lactobacillus casei*, *L. rhamnosus*, *L. bulgaricus*, *L. acidophilus*, *Bifidobacterium breve*, *B. longum*, *Streptococcus thermophiles*; total viable count, 2 × 10^11^ CFU) on seminal quality, markers of seminal oxidative stress, inflammatory factors and reproductive hormones in men with idiopathic oligoasthenoteratozoospermia. It was found that, compared to controls receiving placebo, males who received probiotics for 10 weeks showed higher seminal volume, sperm concentration, and number of spermatozoa, and motile sperm. In rats fed with a high-fat diet [32], probiotic supplementation (based on *Lactobacillus* spp., *Bacillus* spp., beer yeast, and photo-synthetic bacteria culture) reduced sperm lipid peroxidation and enhanced sperm motility, viability, integrity, and sperm count. In obesity-induced male mice, the administration of *L. rhamnosus* PB01 enhanced sperm quality and sex hormone levels [33].

The results from all these studies, in which increased sperm quality after probiotics was observed, differ from ours perhaps because such studies were based on individuals who initially had poor semen quality, whereas, in our case, our control group consisted of healthy individuals. Thus, a positive action of probiotics is evident in individuals with poor-quality samples. Furthermore, the duration of administration differed from that used in the present study. The combination of different strains of *Lactobacillus* with *Bifidobacterium* and *Streptococcus* (Vivomixx®) or with *F. duncaniae* may also be the reason why sperm parameters are maintained. *F. duncaniae* is the most abundant bacteria in gut microbiota and has beneficial properties to the host because it can ferment glucose into short-chain fatty acids, being the most important bacteria in producing butyrate in the human colon [15]. Nevertheless, to the best of our knowledge, no studies have evaluated its effects on sperm traits, perhaps because of the difficulties for culture, as it is a strictly anaerobic bacterium. One aspect to be considered is the presence of inulin in the culture media of *F. duncaniae* since it has been described that it could be beneficial for sperm traits in diabetes-induced rats [43] when combined with *Lactiplantibacillus plantarum*. The residual presence of this fructan in the probiotic administered could have some effects on sperm traits that need to be addressed further.

In the present study, the combination of probiotics increased the percentage of normal sperm, decreasing abnormalities in the head. Differences in sperm nuclear morphology also arose, with a smaller area, minimum diameter, head body width, and circularity in the sperm of individuals treated with probiotics, resulting in apparently less rounded (more elongated) spermatozoa. These differences may be the reason for the higher linearity in the probiotic group A (*L. rhamnosus* and *F. duncaniae*) after 60-min incubation, which is an indicator of sperm survival in conditions similar to those found in the female tract, suggesting an improvement in sperm swimming during the journey to the egg. These more elongated sperm also showed higher velocities (VCL, VSL), although not statistically different from controls, in accordance with previous studies that report that longer sperm swim faster [41, 44].

While the results presented here suggest that probiotics may be beneficial for semen quality, there are limitations in this study that need to be addressed in the future. A larger scale trial, with a larger sample size and different probiotic concentrations, may lend further strength and support to the effects we observed. In addition, a longer time of exposure to probiotics may reveal further beneficial effects on other sperm parameters. Here, administration of probiotics for 5 weeks was chosen, taking into account the length of one spermatogenic cycle and the time spermatozoa transit along the epididymis in the mouse [34–37]. This approach agrees with the effects seen after the supplementation for just one spermatogenic cycle in zebrafish [30]. Interestingly, extending the period of supplementation of probiotics in zebrafish did not appear to improve the results beyond those seen earlier [31]. Studies in dogs, horses, and humans vary in the length of treatment (3 weeks to 6 months), which may relate to the success or failure of improvements in seminal parameters [26–29, 45]. Long administration periods may be feasible in mice with an earlier start in treatment, examining effects from puberty onwards. Alternatively, administration for longer periods in adult individuals needs to bear in mind the onset of senescence and additional potential side-effects extending further in adult life [46–48]. To gain additional information on the impact of probiotics on male reproduction, sperm functional tests may shed light on the possible effects of probiotics on capacitation, hyperactivation, and induced acrosomal exocytosis [49]. Since sperm metabolism is crucial to sustain these functions, further characterization of probiotic effects will benefit from the characterization of metabolic pathways and metabolite quantification under the various physiological conditions attending fertilization [40]. It may also be of great interest to examine possible confounding variables such as diet variations (volume of food consumed), stress, environmental conditions, or animal handling [50, 51]. Finally, it is generally accepted that sperm parameters are important determinants of male fertility; in particular, the proportion of normal sperm, percentage of motile sperm, and descriptors of sperm kinematics correlate well with pregnancy rates and litter size [49]. Therefore, further studies need to be designed to test in a more direct fashion the fertility of males receiving the various probiotics as well as any effects that may arise in the offspring.

In conclusion, the administration of a mixture of *L. rhamnosus* and *F. duncaniae*, as well as the commercial probiotic Vivomixx®, seems not to be detrimental to sperm quality and, in fact, may exert a beneficial effect on sperm normal morphology when administered for five weeks. This study opens new perspectives on the assessment of probiotic effects, but additional studies will be required to examine the effects of more prolonged administration in a higher number of individuals and the possible beneficial effects on other sperm traits important for fertilization.

## Supplementary Information

Below is the link to the electronic supplementary material.

Supplementary file1 (DOCX 671 KB)

## Data Availability

Data are provided within the manuscript and supplementary information files.
