# Supplementary material for: Effect of Probiotics on Sperm Quality in the Adult Mouse
Source: Probiotics Antimicrob Proteins. 2024 Oct 23;17(6):4480–90. doi: 10.1007/s12602-024-10388-z (PMC12634785; doi:10.1007/s12602-024-10388-z)
Supplement: Supplementary file 1 — Supplementary file1 (DOCX 671 KB) [file 12602_2024_10388_MOESM1_ESM.docx]

**Supplementary Material for**

**Effect of probiotics on sperm quality in the adult mouse**

Ana Sanchez-Rodriguez^1^, Ingrid I. D. Idrovo^1^, Rocío Villafranca^1^, Nerea Latorre^1^, Juan Antonio Rielo^1^, Ane Laburu^2^, Sandra Nieto Román^2,3^, Daniel Heredia^3^, Rubén González^3^, Virginia García-Cañas^4^, Diego Laxalde^4^, Carolina Simó^4^, David R. Vieites^5^, Eduardo R. S. Roldan^1^*

^1^Department of Biodiversity and Evolutionary Biology, Museo Nacional de Ciencias Naturales (CSIC), Calle José Gutierrez Abascal 2, 28006-Madrid, Spain.

**^2^**Department of Biogeography and Global Change, Museo Nacional de Ciencias Naturales (CSIC), Calle José Gutierrez Abascal 2, 28006-Madrid, Spain.

^3^BioCoRe S. Coop. Calle Primitiva Gañan 11, 28026, Madrid, Spain.

^4^Molecular Nutrition and Metabolism, Institute of Food Science Research (CSIC), Calle Nicolás Cabrera 9, 29049-Madrid, Spain.

^5^Institute of Marine Research (CSIC), Rúa Eduardo Cabello 6, 36208-Vigo, Spain.

*****Correspondence: roldane@mncn.csic.es

**Contents**

**Fig. S1.** Segments in mouse sperm nuclei identified by morphology analysis.

**Fig. S2.** Parameters used for analysis of sperm nuclear morphology.

**Fig. S3.** Effect of probiotics on body measurements in adult mice.

**Fig. S4.** Effect of probiotics on sperm concentration and total number of sperm in adult mice.

**Fig. S5.** Comparison of the interquartile range (IQR) profiles between sperm nuclei from untreated controls, probiotic V, and probiotic A.

**Fig. S6.** Comparison of angle profiles of sperm nuclei from untreated controls, probiotic V and probiotic A.

**Table S1**. Size and shape measurements of spermatozoa from controls and from groups treated with different probiotics.


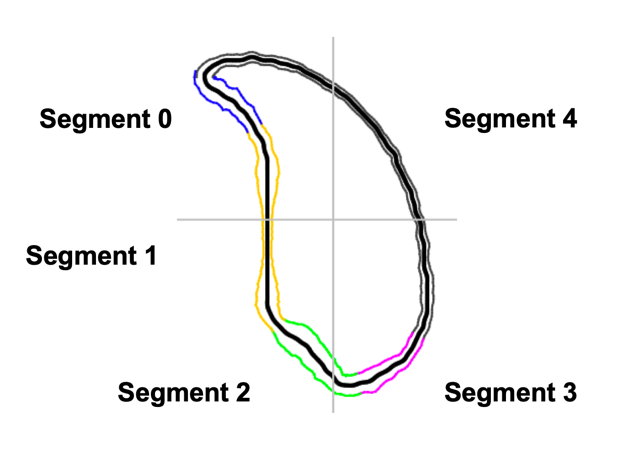


**Fig. S1.** Segments in mouse sperm nuclei identified by morphology analysis [38]. Segment 0 is shown in dark blue, segment 1 in yellow, segment 2 in light green, segment 3 in purple, and segment 4 in black.

**
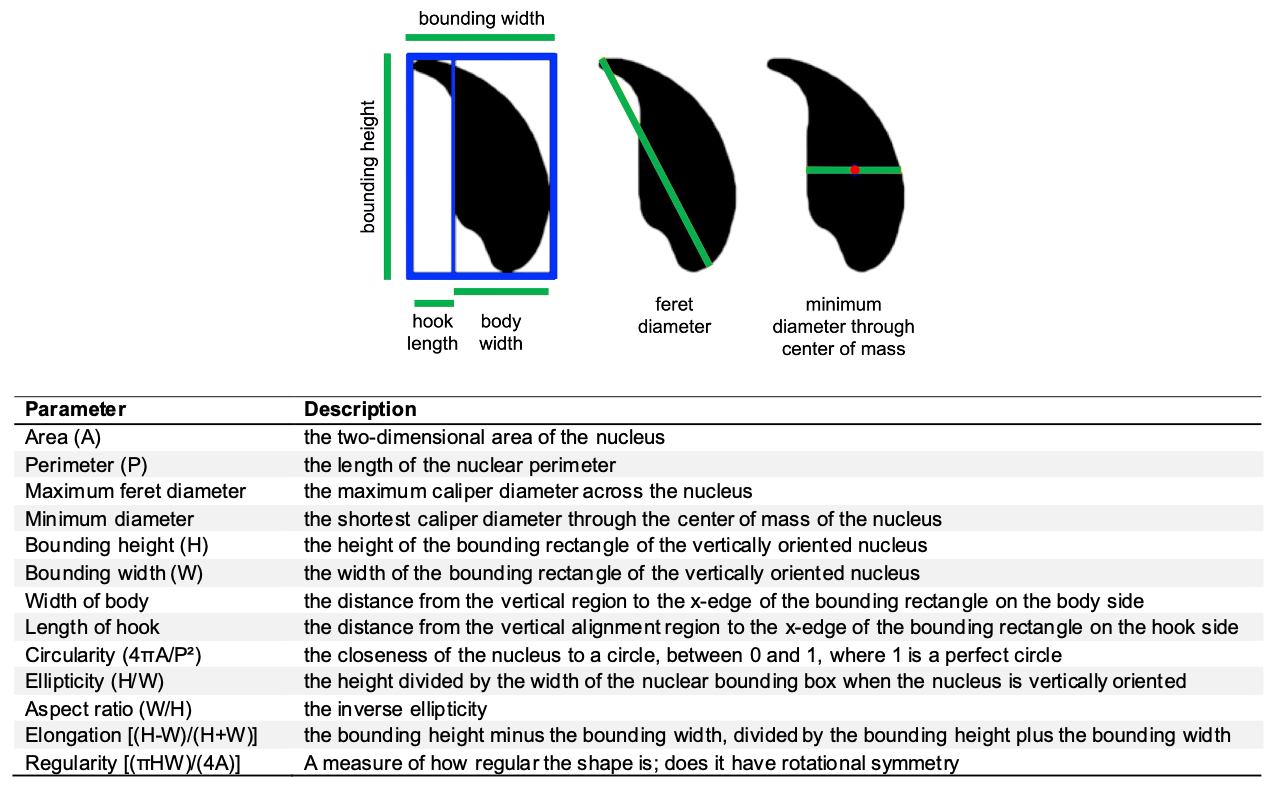
**

**Fig. S2.** Parameters used for analysis of sperm nuclear morphology [38].

**Fig. S3.** Effect of probiotics on body measurements in adult mice. **A**, body mass; **B**, testes mass; **C**, relative testes mass (RTS). No significant differences were observed (*p*>0.05). Data are means ± SEM.

**Fig. S4.** Effect of probiotics on sperm concentration and total number of sperm in adult mice. **A**, sperm concentration; **B**, total number of spermatozoa. Spz: sperm. No significant differences were observed (*p*>0.05). Data are means ± SEM.

**
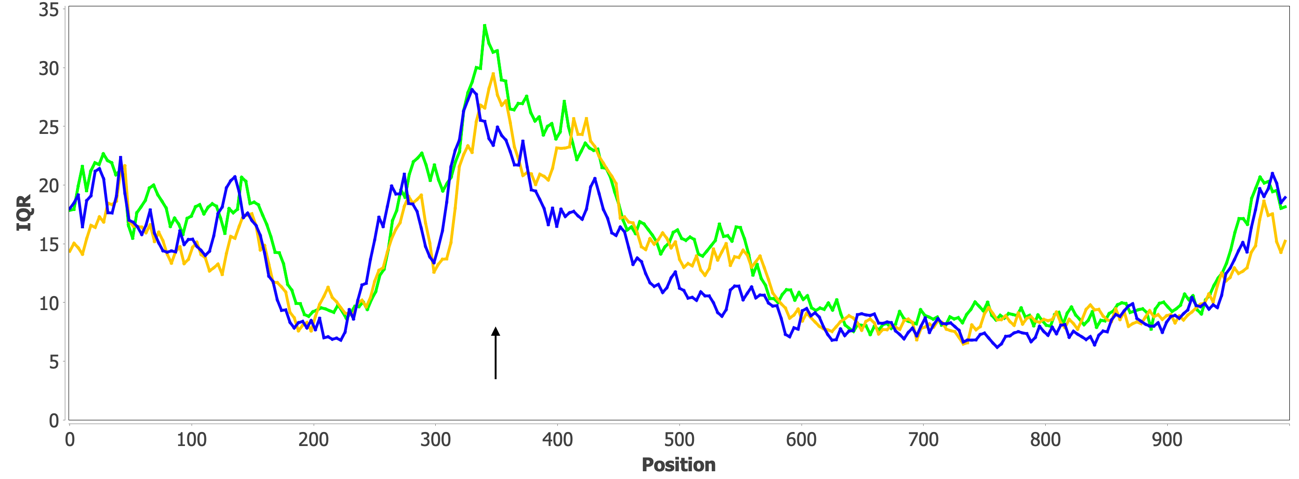
**

**Fig. S5.** Comparison of the interquartile range (IQR) profiles between sperm nuclei from untreated controls (blue), probiotic V (yellow), and probiotic A (green). Black arrow indicates greater dispersion at position 350 that corresponds to segment 3.


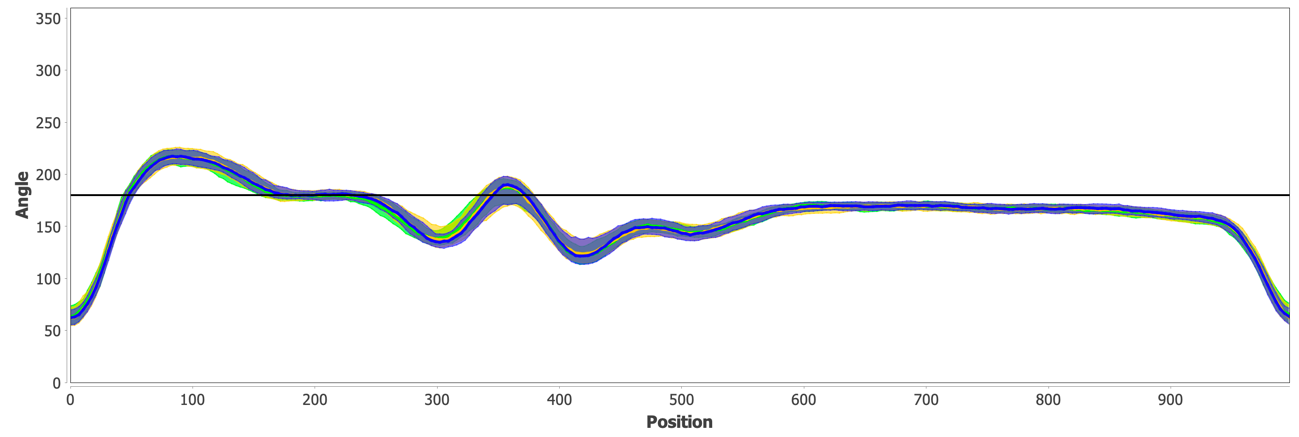


**Fig. S6.** Comparison of angle profiles of sperm nuclei from untreated controls (green), probiotic V (blue) and probiotic A (yellow) showing the median and interquartile range of the nuclear angle profiles.

**Table S1**. Size and shape measurements of spermatozoa from controls and from groups treated with different probiotics. Different letters in the same row indicate significant differences when compared to controls (*p*<0.05). Data are mean ± SEM.

| **Parameter** | **Control** | **Group V** | **Group A** |
| --- | --- | --- | --- |
| Area (µm^2^) | 21.21 ± 0.17 ^a^ | 20.08 ± 0.16 ^b^ | 20.28 ± 0.16 ^b^ |
| Perimeter (µm) | 21.4 ± 0.17 | 21.44 ± 0.16 | 21.69 ± 0.17 |
| Maximum feret (µm) | 7.93 ± 0.04 | 7.85 ± 0.03 | 7.94 ± 0.05 |
| Minimum diameter (µm) | 3.43 ± 0.03 ^a^ | 3.28 ± 0.03 ^b^ | 3.26 ± 0.03 ^b^ |
| Bounding height (µm) | 7.23 ± 0.06 | 7.19 ± 0.04 | 7.23 ± 0.06 |
| Bounding width (µm) | 5.22 ± 0.06 | 5.09 ± 0.04 | 5.12 ± 0.05 |
| Width of body (µm) | 3.79 ± 0.07 ^a^ | 3.56 ± 0.05 ^b^ | 3.6 ± 0.05 ^b^ |
| Length of hook (µm) | 1.47 ± 0.06 | 1.57 ± 0.05 | 1.55 ± 0.05 |
| Circularity (4πA/P²) | 0.59 ± 0.01 ^a^ | 0.56 ± 0.01 ^ab^ | 0.55 ± 0.01 ^b^ |
| Ellipticity (H/W) | 1.42 ± 0.02 | 1.44 ± 0.02 | 1.45 ± 0.02 |
| Aspect ratio (W/H) | 0.74 ± 0.02 | 0.72 ± 0.01 | 0.72 ± 0.01 |
| Elongation [(H-W)/(H+W)] | 0.16 ± 0.01 | 0.17 ± 0.01 | 0.17 ± 0.01 |
| Regularity [(πHW)/(4A)] | 1.39 ± 0.01 | 1.44 ± 0.01 | 1.43 ± 0.01 |
